# The complete mitochondrial DNA of *Ancherythroculter wangi*

**DOI:** 10.1080/23802359.2017.1413320

**Published:** 2017-12-13

**Authors:** Taiming Yan, Xiongyan Wang, Songpei Zhang, Liang He, Zhi He

**Affiliations:** College of Animal Science and Technology, Sichuan Agricultural University, Chengdu, China

**Keywords:** *Ancherythroculter wangi*, mitochondrial genome, phylogenetic analyses

## Abstract

The complete mitochondrial genome of *Ancherythroculter wangi* was determined. The mitochondrial genome, consisting of 16,622 base pairs (bp), encoded 13 protein-coding genes, 2 ribosomal RNAs, 22 transfer RNAs and a non-coding control region, as those found in other *Ancherythroculter* species. These results can provide useful data for further studies on taxonomic status, molecular systematics, and stock evaluation.

*Ancherythroculter wangi* belongs to the family Cyprinidae and the order Cypriniformes, and mainly distributed in the upper stream of Yangtze River and its tributary (Ding 1994; Yue et al. 2000). There was less report about its basic biology data including genetic information. In this study, we first determined the complete mitochondrial genome of *A. wangi*, which provides the basic molecular data for the further study on its systematics and conservation biology.

In the present study, one specimen *A. wangi* chosen for mitochondrial genome analysis were collected from the downstream of Qi River (N:30°39.534’, E:105°11.893’) (specimen is stored in Aquaculture Department of Sichuan Agricultural University, number Aw2017092801). Primers were designed for polymerase chain reaction (PCR) amplification and sequencing on the basis of the mitogenome sequence of *Ancherythroculter lini* (GenBank accession no. NC_027741) (Chen et al. 2016). The complete mt genome of *A. wangi* was 16,621 bp and has been deposited in GenBank with accession no. MG575902. The mitochondrial genome encoded 13 protein-coding genes, two ribosomal RNAs, 22 transfer RNAs and a non-coding control region, as those found in other *Ancherythroculter* species (Wan et al. 2013; Chen et al. 2016; Wang et al. 2016). The nucleotide composition of the genome of *A. wangi* is A 31.2%, T 24.9%, G 16.2% and C 27.7%. Except for the nad6 and eight tRNA genes (*tRNA-Gln*, *tRNA-Aln*, *tRNA-Asn*, *tRNA-Cys*, *tRNA-Tyr*, *tRNA-SerUCN*, *tRNA-Glu* and *tRNA-Pro*) encoded on the light-strand, all others genes were encoded on the heavy-strand. This is a typical gene arrangement conforming to the other *Ancherythroculter* species and vertebrate consensus (Wan et al. 2013; Chen et al. 2016; Wang et al. 2016).

All genes use ATG as start codon, except *cox1* use GTG, which also discovered in other *Ancherythroculter* species (Wan et al. 2013; Chen et al. 2016; Wang et al. 2016). Most open reading frames ended with two types of complete stop codons TAA and TAG, whereas the other seven genes, *ND2*, *COX2*, *ATP8*, *COX3*, *ND3*, *ND4* and cytochrome b (CYTB), had an incomplete termination codon T or TA.

Based on combined nucleotide sequence data of 12 heavy-strand protein-coding genes of *A. Wangi*, and together with the sequences of other *Ancherythroculter species*, phylogenetic trees were constructed by using the UPGMA methods (Figure 1). All *Ancherythroculter species* had close relationship, *A. Wangi*, *A. kurematsui* and *A. nigrocaudai* were monophyletic in the trees. Numbers on the nodes correspond to bootstrap values based on 1000 iterations. Thus, the mitochondrial genome data and phylogenetic analysis of the *A. Wangi* can enrich the basic molecular data of *Ancherythroculter*.

**Figure 1.**
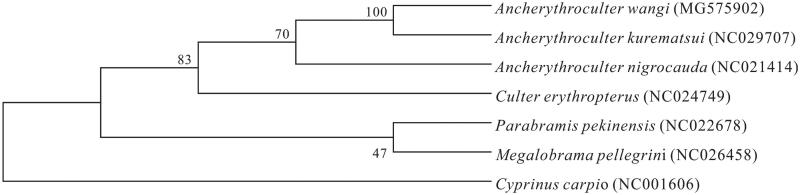
Phylogenetic relationships among three species of *Ancherythroculter* inferred from UPGMA of deduced amino acid sequences of 12 mitochondrial proteins. The numbers on the branches are bootstrap values for UPGMA.

## References

[CIT0001] Chen Y, Hu J, Zhao H, Yang H, Liu L. 2016 The complete mitochondrial genome of the *Ancherythroculter lini* (Cypriniformes, Cyprinidea). Mitochondrial DNA A DNA Mapp Seq Anal. 27:3043–3044. 27158794 10.3109/19401736.2015.1063126

[CIT0002] Ding RH. 1994 The fishes of Sichuan, China. Chengdu (China): Sichuan Publishing House of Science and Technology pp. 203–205.

[CIT0003] Yue P, Shan X, Lin R. 2000 Fauna sinica: osteichthyes cypriniformes III. Beijing (China): Science Press.

[CIT0004] Wan Q, Chen Y, Cheng Q, Qiao H. 2013 The complete mitochondrial genome sequence of *Ancherythroculter nigrocauda* (Cypriniformes:Cyprinidae). Mitochondrial DNA. 24:627–629. 23452280 10.3109/19401736.2013.772157

[CIT0005] Wang GJ, Zheng ZL, Yu EM, Xie J, Wei N, Wu JR, Li JS. 2016 The complete mitochondrial genome of *Ancherythroculter kurematsui* (Cypriniformes: Cyprinidae). Mitochondrial DNA Part B. 1:630–631. 10.1080/23802359.2016.1214547 PMC7800160 33473577

